# Agreement between self-reported perinatal outcomes and administrative data in New South Wales, Australia

**DOI:** 10.1186/s12884-015-0597-x

**Published:** 2015-08-04

**Authors:** Ellie Gresham, Peta Forder, Catherine L. Chojenta, Julie E. Byles, Deborah J. Loxton, Alexis J. Hure

**Affiliations:** Research Centre for Gender, Health and Ageing, School of Medicine and Public Health, University of Newcastle, and Hunter Medical Research Institute, Newcastle, Australia

## Abstract

**Background:**

Many epidemiological studies that focus on pregnancy rely on maternal self-report of perinatal outcomes. The aim of this study was to evaluate the agreement between self-reported perinatal outcomes (gestational hypertension with or without proteinuria, gestational diabetes, premature birth and low birth weight) in a longitudinal study and linked to administrative data (medical records).

**Methods:**

Self-reported survey data from the Australian Longitudinal Study on Women’s Health was linked with the New South Wales Perinatal Data Collection. Agreement between the two sources was evaluated using percentage agreement and kappa statistics. Analyses were conducted at two levels by: i) the mother and ii) each individual child.

**Results:**

Women reliably self-report their perinatal outcomes (≥87 % agreement). Gestational hypertension with or without proteinuria had the lowest level of agreement. Mothers’ reports of perinatal outcomes were more reliable when evaluated by child. Restricting the analysis to complete and consistent reporting further strengthened the reliability of the child-specific data, increasing the agreement from >92 to >95 % for all outcomes.

**Conclusions:**

The present study offers a high degree of confidence in the use of maternal self-reports of the perinatal outcomes gestational hypertension, gestational diabetes, preterm birth and low birth weight in epidemiological research, particularly when reported on a per child basis. Furthermore self-report offers a cost-effective and convenient method for gathering detailed maternal perinatal histories.

## Background

Many epidemiological studies that focus on pregnancy rely on maternal self-report to obtain information on perinatal events. Self-report is a cost-effective, efficient method to collect perinatal information [1], however, it is important to know the reliability of these reports [2]. Administrative data (data obtained from medical records) have the advantage of being routinely collected and can be used for monitoring maternal and perinatal outcomes [3]. In this study, administrative data includes birth records collected from both public and private hospitals, and private midwifery or medical practitioners who deliver babies outside the hospital for all births in New South Wales (NSW). Obtaining such information from medical records can be expensive, time-consuming, and may contain data inaccuracies, such as incorrect data entry due to incomplete, inaccurate or missing diagnoses [4, 5]. Administrative data are also limited in the information recorded, subsequently restricting the research questions that can be answered.

There is debate throughout the literature as to whether administrative (medical) records can be used as a gold standard for comparing data and accuracy may vary depending on the exact data source [2, 6–11]. Evidence indicates that combining both self-report and administrative data may provide the most valid and complete assessment [2, 12–16]. Recent analysis of data from the Australian Longitudinal Study on Women’s Health (ALSWH) found that no perinatal data source was entirely accurate or reliable [2]. That study assessed the validity of self-reported stillbirth data using three state-based administrative records (the Perinatal Data Collection (PDC); Admitted Patient Data Collection; and Perinatal Death Review Database). Overall the administrative datasets performed better for validity than the self-reported data for confirmed cases of stillbirth, with the PDC showing 90 % sensitivity and 96 % specificity compared to the self-reported stillbirth data: 100 % sensitivity but only 30 % specificity. Hure et al. concluded that self-reported stillbirth data have the advantage of being readily available and may provide much more information than any single administrative dataset, but caution needs to be applied when cross-checking, externally validating and data cleaning so data are used appropriately.

A number of other studies in the United States [7, 11, 17, 18], United Kingdom [4, 6], Canada [19], France [20] and the Netherlands [21] have measured the agreement of maternal self-reported perinatal outcomes and official health records. The majority of these agreement studies have focused on maternal recall of birth weight, gestational age and mode of delivery. Agreement between maternal self-report and medical records is high for low birth weight (kappa 0.82-0.87) [4, 19, 21]. Moderate to high agreement has been shown for premature birth (kappa 0.41-0.87) [17–19, 21]. While, the pregnancy outcomes gestational hypertension and gestational diabetes have been reported less frequently [7, 11, 21], with varying degrees of agreement (gestational hypertension kappa 0.59-0.68; gestational diabetes kappa 0.40-0.83).

Worldwide, there have been a number of studies that assess the accuracy of maternal self-report for perinatal outcomes in comparison to administrative data, with one study conducted in Australia examining a single perinatal outcome (stillbirth). This study aims to extend the published literature, examining the agreement in an Australian setting between self-reported perinatal outcomes (gestational hypertension with or without proteinuria, gestational diabetes, premature birth and low birth weight) and health records (administrative data). Self-reported data were obtained from the ALSWH, linked with the state PDC, which provides a minimum data set for perinatal outcomes research. The objectives of this study were to: i) determine the agreement between self-report and PDC data by the mother and for each individual child, ii) evaluate the reliability of maternal reports longitudinally using child-specific survey data and iii) identify the implication for practice findings of using either data source.

## Methods

### Self-reported survey data

#### The Australian Longitudinal Study on Women’s Health (ALSWH): 1973–1978 cohort

The ALSWH was established to examine demographic, social, physical, psychological, and behavioural variables associated with women’s health, well-being and health service usage. Full details of recruitment have been published elsewhere [22–24]. Briefly, in 1996, over 40,000 women were recruited in three age cohorts: born 1973–78 (18–23 years), 1946–51 (45–50 years) and 1921–26 (70–75 years). Participants were randomly selected from Australia’s universal health insurance database (Medicare), with intentional oversampling of women in rural and remote areas [24]. Ethics approval for the ALSWH were obtained from the Human Research Ethics Committees of the Universities of Newcastle (H-076-0795) and Queensland (2004000224), and written informed consent was provided by participants.

This study examines data from the 1973–78 cohort, who were broadly representative of the Australian population at the baseline survey [23]. Paper-based surveys were mailed to 14,247 participants in 1996 (Survey 1), 2000 (Survey 2), 2003 (Survey 3), 2006 (Survey 4), 2009 (Survey 5), and 2012 (Survey 6). Pregnancy and birth data were collected at each survey. Survey 6 responses were received from 8010 women, with 25 % (*n* = 1,952) residing in NSW at that time [25, 26].

#### Pregnancy outcomes

At each survey from Survey 2 to Survey 4 women were asked to recall whether they had been told by a doctor or treated for the conditions of 'Hypertension (high blood pressure) during pregnancy' or 'Gestational diabetes' with response options of ‘yes’ and ‘no’. For Surveys 5 and 6, women were asked the same questions with respect to each of their individual children (1^st^ child, 2^nd^ child etc.).

#### Birth outcomes

Premature birth data were collected from Survey 2 onwards. For Surveys 2–4, women were asked ‘How many times have you had each of the following?’ with ‘Live premature birth (36 weeks or less)’ one of the pregnancy outcomes. Response categories were ordinal (‘1’, ‘2’, ‘3’, ‘4’ and ‘5 or more’). From Survey 5, women were asked ‘Did you experience any of the following?’ with ‘Premature birth’ in the list of perinatal outcomes (yes/no), reported for each of the woman’s children with no gestational cut-off specified.

Low birth weight data has been collected since Survey 4. For each child, women were asked ‘Did you experience any of the following?’ with ‘A low birth weight baby (weighing less than 2500 g or 5 ½ pounds)’ listed as one of the pregnancy outcomes (yes/no).

### Medical records

#### Perinatal Data Collection (PDC)

The PDC was developed in 1986 [27] and provides information on pregnancy care, and maternal and newborn outcomes as recorded by the attending midwife or medical practitioner [28, 29]. In NSW, all live births and stillbirths of at least 20 weeks gestation or at least 400 g birth weight are recorded in the PDC [28]. For multiple births, a separate form is completed for each baby [29]. Demographic, medical and obstetric information are collected on the mother, as well as information on the labour, delivery and condition of the baby [29]. PDC does not receive notifications of interstate births when the mother is usually a resident in NSW [28].

#### Pregnancy outcomes

Gestational diabetes, pregnancy-induced/gestational hypertension and preeclampsia are binary coded as either occurring in the mother or not (yes/no). Over time, multiple terms were used to classify gestational hypertension and preeclampsia. In 1994, pregnancy-induced hypertension included women with either pregnancy-induced hypertension or preeclampsia. In 2006, pregnancy-induced hypertension was separated into women with proteinuric and non-proteinuric pregnancy-induced hypertension, and in 2011, while the coding remained consistent (yes/no), the wording changed to gestational hypertension (non-proteinuric) and preeclampsia (proteinuric).

#### Birth outcomes

Gestational age and birth weight are reported in the PDC as continuous variables, in weeks and grams respectively.

### Data linkage

Data linkage was performed in May 2014 by the Centre for Health Record Linkage (CHeReL, www.cherel.org.au), independent of the researchers and data custodians. The data linkage used probabilistic matching and *ChoiceMaker* software [30] for the two data sources. The following identifiers: surname, alternative surname, given names, sex (female for all cases), date of birth, age in years, address, locality, postcode, country of birth and notification date were used for matching, which covered the period from January 1, 1994 to 31 December 2011. Children born before (*n* = 315) or after (*n* = 410) these dates were not included in the analysis. Data from a total of 2,446 women in NSW (92.8 % of the 1973–78 ALSWH cohort) were linked with NSW PDC records, with a 0.5 % false positive rate (5/1,000) [31]. Researchers were provided anonymised linked data.

ALSWH used opt-out consent for data linkage. That is, all women who provided written informed consent to participate in the 1973–78 ALSWH cohort were included in data linkage, unless they indicated at any time that they did not want their survey records linked with administrative data such as the PDC. Ethics approval for data linkage were received from the NSW Population and Health Services Research Ethics Committee (2010/06/244), and approval registered with the University of Newcastle.

### Combining survey and PDC data

Self-reported survey data (ALSWH) were collected from 1 July 1996 to 30 November 2013, spanning 17 years of prospective data collection. PDC data were available from 1 January 1994 to 31 December 2011 for 2,631 women who had at least one record in the PDC. The perinatal outcomes included in these analyses are listed in Table 1.

**Table 1:** Perinatal outcomes in New South Wales, Australia: timelines for linked self-reported and administrative datasets

Datasets and outcomes	ALSWH surveys	
Australian Longitudinal Study on Women’s Health, NSW only^a^		
Gestational hypertension	2-6	
Gestational diabetes	2-6	
Premature birth		
36 weeks or less	2-4	
No specified gestational cut-off	5-6	
Low birth weight	4-6	
	Data from	To
Perinatal Data Collection		
Hypertensive disorders^b^		
Pregnancy induced hypertension or preeclampsia	1 January 1994	31 December 2005
Pregnancy induced hypertension – proteinuria	1 January 2006	31 December 2010
Pregnancy induced hypertension – non proteinuria	1 January 2006	31 December 2010
Gestational hypertension	1 January 2011	31 December 2011
Preeclampsia	1 January 2011	31 December 2011
Gestational diabetes	1 January 1994	31 December 2011
Gestational age	1 January 1994	31 December 2011
Birth weight	1 January 1994	31 December 2011

*ALSWH* Australian Longitudinal Study on Women’s Health

^a^Includes all participants who answered ≥1 Australian Longitudinal Study on Women’s Health survey and had at least one record in the Perinatal Data Collection

^b^Hypertensive disorders were the collective term used to classify gestational hypertension and preeclampsia as multiple terms were used over time

#### Pregnancy outcomes

Gestational hypertension reported in the survey data were compared against a pooled hypertensive variable (that included pregnancy-induced hypertension, gestational hypertension and preeclampsia) in the PDC as multiple terms were used over time. These pooled conditions are all characterised by hypertension (≥140 mmHg systolic or ≥90 mmHg diastolic) after 20 weeks gestation [32].

#### Birth outcomes

The most common clinical definition of premature birth is <37 completed weeks gestation [32, 33]. However, due to discrepancies in the ALSWH survey definitions two categories were used to define premature birth according to gestational age: (i) 36 weeks or less for births that occurred before Survey 5 where 36 weeks was specified as the gestational cut-off in the ALSWH surveys and (ii) less than 37 completed weeks for Surveys 5 and 6. Gestational age in the PDC were re-coded categorically to match the relevant cut-point to enable comparison between survey (categorical) and PDC (continuous) data.

Birth weights <2500 g is classified as low birth weight [32]. Birth weights in the PDC were re-coded categorically to enable comparison of low birth weight between survey (categorical) and PDC (continuous) data.

### Statistical analysis

Analyses were performed using Stata IC, version 13 (StataCorp, USA) [34]. Agreement between the self-reported survey and PDC data were evaluated using percentage agreement and kappa statistics. The kappa statistic measures the agreement between the self-reported survey and PDC data with respect to the perinatal outcomes, after accounting for chance agreement [35]. Kappa values > 0.75 indicate excellent agreement, 0.75 to 0.40 moderate agreement and < 0.4 poor agreement [35].

Analyses were performed separately (i) by mother and (ii) per child, since each mother could have more than one birth. To determine how reliably women self-report, data were analysed by mother at first-report (cross-sectional) and pooled across all children to generate ‘ever experienced’ for the perinatal outcomes for both survey and PDC data.

Reliability of the self-report and PDC data were further investigated per child. Multiple births and siblings were included in the analysis, with each child treated as a single unit of analysis. Children were matched according to date of birth and specific project identifier. Agreement was assessed at i) first-report (cross-sectional) and ii) subsequently measured across surveys (for complete and consistent reporting), to correct for inconsistent reporting over time. Complete reporting was defined as the mother completing all surveys reported on a per child basis (Surveys 4–6 for low birth weight, and Surveys 5–6 for gestational hypertension with or without proteinuria, gestational diabetes and premature birth (less than 37 weeks only)). Consistent reporting was defined as the mother reporting the same response (either ‘yes’, ‘no’ or ‘missing’) at each survey for each child, with outcomes reported on a per child basis.

## Results

**Fig. 1:**
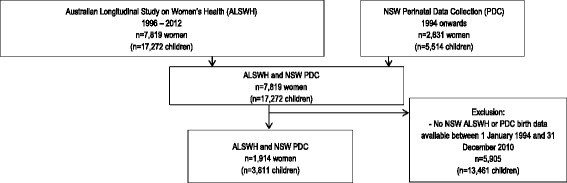
Flow chart showing the selection of participants, using the 1973–1978 cohort from the Australian Longitudinal Study on Women’s Health (ALSWH) and the NSW Perinatal Data Collection (PDC)

The selection of participants eligible for this study is presented in Fig. 1. Of the 1,914 women with linked perinatal data, the frequency of perinatal outcomes were self-reported across the ALWSH surveys and recorded by a midwife or attending medical practitioner in the PDC data respectively as: gestational hypertension with or without proteinuria, 336 (18 %) versus 229 (12 %); gestational diabetes, 226 (12 %) versus 86 (4.5 %); premature birth, 280 (15 %) versus 169 (9 %); and low birth weight baby 164 (9 %) versus 136 (7 %).

Table 2 contains baseline characteristics of women in the ALSWH 1973–78 cohort who were included in the study (NSW only) and for those who did not have linked perinatal data (the other women in the ALSWH cohort). There were no statistically significant differences observed between women included and not included in the study in regard to drinking patterns or available income. However, women included in the study were marginally older than women not included (20.9 vs. 20.7 years respectively; *p* ≤ 0.001), and more lived in an urban area (57.7 % vs. 54.8 %; *p* ≤ 0.001), had no children at baseline (78.8 % vs. 82.2 %; *p* = 0.01). The women were also more likely to be in a partnered (i.e. married or *de facto*) relationship (*p* ≤ 0.01), and were less likely to smoke (*p* = 0.02). While there were a similar number of women who attained school or high school education, there were slightly more women included in the study reporting university education (12.4 % vs. 10.9 %; *p* ≤ 0.01). These women were also more likely to respond at each ALSWH survey, with 75.9 % of women included in the study completing Survey 6 (*p* ≤ 0.001).

**Table 2:** Baseline characteristics of women with linked perinatal data (NSW only, *N* = 1914) and the other women without linked data (ALSWH, *N* = 12333) from the Australian Longitudinal Study on Women’s Health 1973–78 cohort

	Included in the agreement study (NSW only)		Record not linked with PDC (the other women in the ALSWH cohort)	
Characteristic^a^				
Age***	20.9 years		20.7 years	
	n	%	N	%
Number of respondents at each survey***				
1	1914	100.0	12333	100.0
2	1522	79.5	8166	66.2
3	1570	82.0	7511	60.9
4	1633	85.3	7512	60.9
5	1481	77.4	6719	54.5
6	1452	75.9	6558	53.2
Urban resident***	1105	57.7	6764	54.8
Partnered^b^***	526	27.6	2667	21.7
Difficulty managing on income	1005	52.5	6325	51.5
Education***				
No formal education	41	2.2	367	3.0
School or higher school certificate	1274	66.8	8345	68.1
Trade or diploma	355	18.6	2208	18.0
University or higher university degree	237	12.4	1339	10.9
Parity**				
None	1497	78.8	10062	82.2
One	253	13.3	1308	10.7
Two or more	137	7.2	813	6.7
Smoking Status*				
Never Smoked	965	52.9	6158	52.2
History of Smoking	297	16.3	1788	15.2
Current Smoker	562	30.8	3859	32.7
Alcohol Intake Status				
Non Drinker	164	8.7	1090	8.9
Rarely drinks/low risk drinker	1633	86.5	10419	85.4
Often drinks/high risk drinker	91	4.8	691	5.7

*n* number, *PDC* Perinatal Data Collection

*P-value is statistically significant at ≤0.05

**P-value is statistically significant at ≤0.01

***P-value is statistically significant at ≤0.001

^a^Participant characteristics were taken at baseline when the women were aged 18–23 years (ALSWH survey 1 (1996))

^b^Includes those women who are married or in a *de facto* relationship

### Agreement of pregnancy and birth outcomes: by mother

Linked data were available for 1,914 women (3,811 children). Table 3 shows the percentage agreement and kappa statistics between self-report survey (ALSWH) and administrative (PDC) data by mother, at first report for the perinatal outcomes. There was high agreement (≥89 %) and moderate kappa statistics (0.42-0.66) observed for all outcomes (*p* < 0.001), with premature birth classified as 36 weeks or less (reported at surveys 2–4) having higher agreement, and lower kappa statistics when compared to premature birth classified as less than 37 weeks gestation (reported at surveys 5–6) (*p* < 0.001). Women had marginally better agreement for all perinatal outcomes and slightly higher kappa statistics for gestational hypertension with or without proteinuria, gestational diabetes and low birth weight at first-report (Table 3) compared to ever experienced (Table 4). Despite analyses performing marginally stronger for all outcomes at first-report, kappa statistics were moderate.

**Table 3:** Agreement of perinatal outcomes between self-reported survey data and the Perinatal Data Collection, by mother, at first-report^a^ for women in the Australian Longitudinal Study on Women’s Health 1973–78 cohort (NSW data only)

Agreement of self-report survey data and administrative data						Agreement		
Perinatal outcomes	Y/Y^b^	N/N^c^	Y/N^d^	N/Y^e^	Total n	% Agreement	Kappa	*P*-value
First-report^a^								
Gestational hypertension with or without proteinuria	141	1500	137	63	1841	89.1	0.52	<0.001
Gestational diabetes	59	1665	138	8	1870	92.2	0.42	<0.001
Premature birth								
Live birth, 36 weeks or less^f^	57	1737	63	53	1910	93.9	0.46	<0.001
Live birth, less than 37 weeks^g^	72	1613	107	28	1820	92.6	0.48	<0.001
Low birth weight (<2500 g)	116	1694	84	19	1914	94.6	0.66	<0.001

*N* no, *Y* yes

^a^‘First-report’ data is the first-reported occurrence (‘yes’ or ‘no’)

^b^Y/Y = positive survey/positive administrative

^c^N/N = negative survey/negative administrative

^d^Y/N = positive survey/negative administrative

^e^N/Y = negative survey/positive administrative

^f^Self-report data at surveys 2–4, with premature birth defined as 36 weeks or less

^g^Self-report data at surveys 5–6, with no specified gestational cut-off provided for premature birth classification

**Table 4:** Agreement of perinatal outcomes between self-reported survey data and the Perinatal Data Collection, by mother, ever experienced^a^ for women in the Australian Longitudinal Study on Women’s Health 1973–78 cohort (NSW data only)

Agreement of self-report survey data and administrative data						Agreement		
Perinatal outcomes	Y/Y^b^	N/N^c^	Y/N^d^	N/Y^e^	Total n	% Agreement	Kappa	*P*-value
Ever experienced^a^								
Gestational hypertension with or without proteinuria	159	1505	177	70	1911	87.1	0.49	<0.001
Gestational diabetes	68	1667	158	18	1911	90.8	0.40	<0.001
Premature birth								
Live birth, 36 weeks or less^f^	57	1134	66	20	1277	93.3	0.54	<0.001
Live birth, less than 37 weeks^g^	98	1197	118	28	1441	89.9	0.52	<0.001
Low birth weight (<2500 g)	89	1641	75	39	1844	93.8	0.58	<0.001

*N* no, *Y* yes

^a^‘Ever experienced’ is the data pooled by mother

^b^Y/Y = positive survey/positive administrative

^c^N/N = negative survey/negative administrative

^d^Y/N = positive survey/negative administrative

^e^N/Y = negative survey/positive administrative

^f^Self-report data at surveys 2–4, with premature birth defined as 36 weeks or less

^g^Self-report data at surveys 5–6, with no specified gestational cut-off provided for premature birth classification

### Agreement of pregnancy and birth data: per child

Linked data were available for 3,811 children (1,914 women). Table 5 presents the percentage agreement and kappa statistics of the comparison between self-reported data in the ALSWH and the PDC for the perinatal outcomes reported per child, at first report. Perinatal outcomes had stronger levels of agreement when analysed per child than by mother. Overall, perinatal outcomes had very high agreement (≥92 %). Gestational hypertension with or without proteinuria was the least reliable at 92 % agreement, while gestational diabetes and low birth weight had the highest percentage agreement (98 %) between survey and administrative data. Kappa statistics were moderate for all outcomes (0.47-0.73). Less than three percent of women reported discrepantly, where self-report differed between surveys for perinatal outcomes.

**Table 5:** Agreement of perinatal outcomes between self-reported survey data and the Perinatal Data Collection for each child born to women in the Australian Longitudinal Study on Women’s Health 1973–78 cohort, at first-report^a^ (NSW data only)

Agreement of self-report survey data and administrative data						Agreement		
Perinatal outcomes	Y/Y^b^	N/N^c^	Y/N^d^	N/Y^e^	Total n	% Agreement	Kappa	*P*-value
First-report^a^								
Gestational hypertension with or without proteinuria	142	2945	161	111	3359	91.9	0.47	<0.001
Gestational diabetes	78	3205	63	12	3358	97.8	0.66	<0.001
Premature birth								
Live birth, less than 37 weeks^f^	155	3051	115	31	3352	95.6	0.66	<0.001
Low birth weight (<2500 g)	124	3459	57	29	3669	97.7	0.73	<0.001

*N* no, *Y* yes, *n* number

^a^‘First-report’ data is the first-reported occurrence (‘yes’ or ‘no’)

^b^Y/Y = positive survey/positive administrative

^c^N/N = negative survey/negative administrative

^d^Y/N = positive survey/negative administrative

^e^N/Y = negative survey/positive administrative

^f^Premature birth defined as less than 37 weeks [32, 33] at surveys 5–6, as no gestational cut-off specified

Approximately 31 % of women had completed surveys 4, 5 and 6 for the outcome low birth weight, while 52 % of women completed surveys 5 and 6 for the outcomes gestational hypertension with or without proteinuria, gestational diabetes and premature birth (less than 37 weeks). Of those women with complete survey responses, more than 50 % of women reported their perinatal history consistently across two (of two) or three (of three) surveys. Perinatal outcomes reported complete and consistently per child (Table 6) were more reliable than outcomes analysed per child at first-report and by mother at first-report or ever experienced (Tables 5 ,3 and 4 respectively). All perinatal outcomes had very high agreement (≥95 %), with variable kappa ranging from moderate to excellent. As for the perinatal outcomes at first-report, gestational hypertension with or without proteinuria was the least reliable, even with an agreement of 94.8 %.

**Table 6:** Agreement of perinatal outcomes between self-reported survey data and the Perinatal Data Collection for each child born to women in the Australian Longitudinal Study on Women’s Health 1973–78 cohort, for complete and consistent reports^a^ (NSW data only)

Agreement of self-report survey data and administrative data						Agreement		
Perinatal outcomes	Y/Y^b^	N/N^c^	Y/N^d^	N/Y^e^	Total n	% Agreement	Kappa	*P*-value
Complete and consistent report^a^								
Gestational hypertension with or without proteinuria	63	1730	49	49	1891	94.8	0.54	<0.001
Gestational diabetes	37	1894	20	5	1956	98.7	0.74	<0.001
Premature birth								
Live birth, less than 37 weeks^f^	60	1807	28	5	1900	98.3	0.76	<0.001
Low birth weight (<2500 g)	30	1099	7	4	1140	99.0	0.84	<0.001

*N* no, *Y* yes, *n* number

^a^‘Complete and consistent report’ includes women who reported the same response (either ‘yes’, ‘no’ or ‘missing’) and completed all surveys (surveys 4–6 for low birth weight, and surveys 5–6 for gestational hypertension with or without proteinuria, gestational diabetes and premature birth (37 completed weeks or less)) for each child

^b^Y/Y = positive survey/positive administrative

^c^N/N = negative survey/negative administrative

^d^Y/N = positive survey/negative administrative

^e^N/Y = negative survey/positive administrative

^f^Premature birth defined as less than 37 weeks [32, 33] at surveys 5–6, as no gestational cut-off specified

## Discussion

We present an agreement study of self-reported survey data (ALSWH) of gestational hypertension with or without proteinuria, gestational diabetes, premature birth and low birth weight with the PDC. Findings suggest that women reliably self-report their perinatal outcomes.

### Interpretation

Mothers’ reports of perinatal outcomes were more reliable when reported on a per child basis, compared to ‘ever experienced’ or ‘first-report’ (children combined by mother). Hence women should be given the opportunity to record their obstetric history for each child rather than as a summary of events. The perinatal outcomes with the highest level of agreement reported by mother were premature birth (36 weeks or less) and low birth weight, while gestational diabetes and low birth weight performed the strongest per child. The high level of agreement associated with gestational diabetes may be due to the mother playing a central role in the control of the condition during her pregnancy, and working closely with health professionals such as her doctor, dietitian and diabetes educator [36]. Premature birth (36 weeks or less, reported by mother only) and low birth weight may have been recalled with greater reliability due to the high social value of the information, with women repeatedly asked to recall their child’s gestational age and birth weight [17]. These items were also defined with birth weight and gestational cut-offs and like gestational diabetes were asked as direct questions.

Gestational hypertension with or without proteinuria and premature birth (less than 37 weeks, reported by mother only) while having good agreement, did not perform as well as the other perinatal outcomes. Lack of communication between the medical practitioner and mother [37], limited understanding of the clinical diagnosis [38], denial [37], or tight control where the mother does not experience any symptoms and is likely to not report the condition, may explain the poorer reliability for gestational hypertension with or without proteinuria. Some confusion may also have viewed preeclampsia as a separate condition to gestational hypertension, rather than on the spectrum of hypertensive disorders of pregnancy. The definition of premature birth has not been consistent within the ALSWH surveys and the wording of this question has been shown to impact on the ability of a woman to provide an accurate response [17]. When asked to report premature birth (reported by mother), women had slightly higher agreement when provided with a definition (93.9 % first-report; 93.3 % ever experienced; *p* < 0.001) than when asked to report the outcome without a gestational cut-off (92.6 % first-report; 89.9 % ever experienced; *p* < 0.001). Despite women knowing their gestational length, they are more likely to over-report premature birth when they are not given a gestational classification.

This paper demonstrated that women self-report perinatal outcomes reliably, with higher agreement when perinatal outcomes are reported per child rather than by mother, even when more time has lapsed between the obstetric event and issue of the survey. Interestingly, there was very little difference in the reliability of perinatal outcomes reported by mother at first-report or ever experienced, indicating that only a small proportion of women are making errors when reporting their perinatal history. Likewise, a small percentage (<3.0 %) of women had discrepant reports per child for gestational hypertension with or without proteinuria, gestational diabetes and premature birth (surveys 5 and 6), and low birth weight (surveys 4, 5 and 6), highlighting that the majority of women report consistently to perinatal outcomes across surveys. Restricting the per child analysis to reports that are complete and consistent, strengthens the reliability, and subsequently increases the agreement from >92 to >95 % for all outcomes.

Birth weight (dichotomised as low birth weight) showed the highest level of agreement in our study. This is consistent with the literature, with the agreement between maternal self-report and administrative data around 90 % [7, 21, 39]. Our finding of more than 95 % agreement for premature birth (reported per child) performed stronger than other studies, which observed 83 % [39], 87 % [7] and 94 % agreement [21]. Our results for gestational diabetes were consistent with the literature [17], while our findings for gestational hypertension with or without proteinuria performed slightly better than other reliability studies [7, 21].

### Implications for practice and research

Our study confirms that maternal reporting of certain perinatal outcomes is highly reliable and can be used in preference to administrative data. Self-report data can be used in epidemiological studies when administrative data are not readily accessible, not obtainable or when the research question requires more depth beyond what is available within administrative datasets [40]. This study is based on a limited number of perinatal outcomes; however, the findings are likely to be applicable to the use of self-reported data that includes any reproductive outcome. Epidemiological researchers when requesting information on premature birth should consider defining and specifying gestational cut-offs for greater reliability.

In longitudinal studies, using first-report is slightly more reliable than pooling data to generate ‘ever experienced’. Therefore, epidemiological researchers with longitudinal data of perinatal outcomes should consider using the mother’s first-report. For greater reliability, using complete and consistent (i.e. responded at all possible surveys and provided the same response at each survey) reporting per child will increase the agreement; however, the small gain in higher agreement may not be warranted as the sample size will subsequently decrease. Any measure that improves data accuracy is important, however, even without data cleaning, the perinatal outcomes gestational hypertension with or without proteinuria, gestational diabetes, premature birth and low birth weight reported in the ALSWH are reliably reported.

Our study has shown that perinatal data reported by mother is reliable; with women making minimal errors. For ALSWH data users, data reported per child (from survey 5) contains the most reliable perinatal data, however using this data in epidemiological research may introduce bias as statistics generally rely on the data being independent i.e. one event/outcome per woman. For women reporting perinatal outcomes, it is reasonable to take the mother’s first-report.

### Limitations

Although the ALSWH is broadly representative of the Australian population, this study was limited to women from NSW only. NSW is the most populous state in Australia, with approximately one-third (32 %) of Australia’s population residing there [41], accounting for 28.9 % (*n* = 4,119) and the largest number of ALSWH participants for the 1973–78 cohort (at baseline). The subsample of women included in the analysis were slightly older, more likely to live in an urban area, more likely to be partnered and university educated and less likely to smoke, therefore caution should be taken when generalising the results to the broader population. Children were excluded from the analysis if they were born after 31 December 2011 (women aged >33 years), with self-report data unable to be matched with PDC records. Despite this limit, there were sixteen years of matchable data for the two datasets, with 93 % of women in NSW in the 1973–78 ALSWH cohort with linked records in the PDC. As with all routinely collected datasets, errors associated with participant recall (outcomes in relation to the timing of the event), data collection, coding and entry are likely to have occurred, including for the PDC. The ALSWH surveys ask women to recall perinatal outcomes, with categorical (yes/no) response options. The PDC record pregnancy outcomes (gestational hypertension with or without proteinuria and gestational diabetes) categorically, while the birth outcomes birth weight and gestational age are recorded as continuous variables. To enable comparison between the two sources, birth outcomes (birth weight and gestational age) in the PDC were recoded into categorical variables. Categorising these outcomes i.e. low birth weight from birth weight has far greater clinical and practical importance in measuring the agreement between the two sources than conducting the analyses to the nearest gram.

From 1994 to 2011, classification of hypertensive disorders were changed multiple times in the PDC, and the diagnostic criteria in clinical practice altered for gestational diabetes. Despite these classification and diagnostic changes, women self-reporting these outcomes are unlikely to know of these intricate details and rely on their medical practitioner for overall diagnosis. Whether epidemiological researchers decide to use self-report or administrative data both incur definitional changes overtime. However, in terms of the agreement, women reliably self-report perinatal outcomes, reinforcing that maternally reported perinatal data can be used in preference to administrative records.

A fundamental strength of this study is the longitudinal nature of the data, which allowed three assessments of the agreement of self-reports of perinatal outcomes with the PDC: (i) responses measured at first-report, (ii) responses pooled across multiple time points ‘ever experienced’ and (iii) responses measured at each survey.

## Conclusions

The present study identifies the strength of using self-reported perinatal outcomes in epidemiological research. The findings offer a high degree of confidence in the use of self-reported data for gestational hypertension with or without proteinuria, gestational diabetes, premature birth and low birth weight outcomes, analysed by mother or per child. The use of questionnaires to measure these outcomes therefore seems justified. However, to further improve the reliability of premature birth, gestational cut-offs should be provided within the question.
